# Preputial Malformation in the Newly Born

**Published:** 1895-09-14

**Authors:** Norman Porritt

**Affiliations:** Hon. Surgeon Huddersfield Infirmary


### MEDICAL PROGRESS AND HOSPITAL CLINICS.

[*The Editor will be glad to receive offers of co-operation and contributions from members of the profession. All letters should be addressed to* THE EDITOR, THE LODGE, PORCHESTER SQUARE, LONDON, W.]

### PREPUTIAL MALFORMATION IN THE NEWLY BORN.

By NORMAN PORRITT, M.R.C.S., L.R.C.P., Hon. Surgeon Huddersfield Infirmary.

It is but rarely that the practitioner is advised to give special attention to the examination of the genital organs in the newly born; and, if the advice has been given, a glance is assumed to be sufficient. It is the mother who monopolises attention throughout the attendance of the accoucheur, the baby being taken possession of by the nurse and its admiring relatives and friends.

An examination of the genitals, and especially of the prepuce, should, however, always be made, for preputial deformities may be the starting-point of more serious secondary affections, which from motives of delicacy may be concealed, whilst, on the other hand, if no serious trouble springs from the abnormal prepuce, it may be necessary at any time after puberty to relieve the now grown-up man by surgical operation. Difficult micturition, retention or incontinence of urine, balanitis, and hernia may follow preputial malformations, not to mention the obstacles to cleanliness and the constant irritation, which may pave the way for masturbation.

The penis of every new-born boy should be taken between the fingers and thumb of the attendant, and the foreskin retracted as far as it will go to ascertain the size of the preputial orifice. The writer's experience is that the phimosis of the text-books—simply an elongated foreskin—is not so common as a foreskin of the natural or less than the natural length, adherent to the glans, and with a mere pin-hole orifice. Troublesome as are the symptoms to which this slight defect gives rise, it will be so hidden by the folds of the foreskin as to escape observation, unless sought for in the manner described.

The symptom which may lead the attendant to suspect this condition is a cross baby. The child has been doctored by the mother or nurse with castor oil and household carminatives with no improvement, and the medical attendant's efforts on similar lines meet with no success. The mother says she gets no rest night or day, and the reason is that the child spends most of its time in repeated, painful attempts to micturate. The mother will say that the child passes plenty of water; his napkins are "never dry," and if they are examined they are rarely found soaking wet, but in a constant state of moistness. I once watched a baby in a paroxysm of pain trying to micturate, and as it screamed a few drops of urine came tardily through the pin-hole orifice in the foreskin, to be followed, after more screaming, by a narrow continuous stream. On operating on the child I found the prepuce and glans glued together in such a manner as to separate the urethral and preputial orifices, so that the urine before escaping had to be forced through the narrow channel between them.

In another case the urinary obstruction set up a comatose condition. Without adequate cause, a fort-night-old baby fell into a condition of semi-stupor. I was puzzled to account for the stupor, for there was no evidence of disease, when the nurse drew my attention to the fact that the child was not passing much water. The foreskin had the pin-hole orifice, and was adherent; and after circumcision was done the child began to pass water freely, and, apparently as a direct result of the operation, recovered its natural liveliness.

Stretching the prepuce has been recommended, but except for slight cases, or where the deformity causes no unpleasant symptoms, circumcision is more satisfactory. Stretching is more often laceration than stretching, and the deformity is more apt to return, for it is difficult to get the mothers to keep the foreskin stretched by retracting it over the glans; many have not the tact to do it, and all are afraid of hurting their latest treasure.

The effects of circumcision are remarkable. Cross, fractious children become quiet. One mother told me that her five months old baby had the best night since it was born the night after the circumcision was done. The peculiar tenderness and painful nature of obstructive urinary disorders in the adult must be borne in mind, and there is no reason to doubt that they are not equally painful in the infant. After the operation the napkins will be either soaking wet or dry, because the child, instead of exhausting itself by the constant attempts to micturate, passes urine in quantity at regular intervals.

On doing circumcision all adhesions between the glans penis and prepuce must be thoroughly broken through, and it is one of the advantages which circumcision has over stretching that it enables this to be done with certainty and precision.

